# The influence of element type and crossed relation on the difficulty of chunk decomposition

**DOI:** 10.3389/fpsyg.2015.01025

**Published:** 2015-07-23

**Authors:** Zhonglu Zhang, Ke Yang, Christopher M. Warren, Guang Zhao, Peng Li, Yi Lei, Hong Li

**Affiliations:** ^1^Research Center of Brain and Cognitive Neuroscience, Liaoning Normal UniversityDalian, China; ^2^College of Educational Science, Chengdu Normal UniversityChengdu, China; ^3^Faculty of Social and Behavioural Sciences, Leiden UniversityLeiden, Netherlands; ^4^Research Centre for Brain Function and Psychological Science, Shenzhen UniversityShenzhen, China

**Keywords:** chunk decomposition, element type, crossed relation, Chinese character

## Abstract

Chunk decomposition is an aspect of problem solving that involves decomposing a pattern into its component parts in order to regroup them into a new pattern. Previous work suggests that the primary source of difficulty in chunk decomposition is whether a problem’s solution requires removing a part that is a meaningful perceptual pattern (termed a chunk) or not (a non-chunk). However, the role of spatial overlap (crossed relation or not) has been ignored in this line of research. Here, we dissociated the role of element type and crossed relation in chunk decomposition problems by employing a Chinese character transformation task. We replicated the finding that when the to-be-removed element is a non-chunk, the problem is more difficult to solve than when the element is a chunk. However, this result held only if the elements had no crossed relation. Relative to non-crossed relation, problems that involved removing elements that overlapped with the remaining character were more difficult to solve irrespective of the element type. We conclude that both element type and crossed relation can cause the difficulty of chunk decomposition and crossed relation plays more important role in preventing people from finding insightful ways to decompose chunk relative to element type.

## Introduction

Creative insight can be conceived of as a sudden solution to a problem which requires restructuring or reorganizing a mental representation of a stimulus, situation, or event to yield non-obvious interpretations (Kounios and Beeman, 2014). It is usually preceded by an impasse that is caused by inappropriate problem representation, and thus needs to be broken or overcome (Ohlsson, 1984; Knoblich et al., 1999; Cranford and Moss, 2012).

How does one break inappropriate problem representations? One crucial way is to change the perceptual structure of the problem, a process described by the chunk decomposition hypothesis (Knoblich et al., 1999, 2001; Luo et al., 2006). According to this hypothesis, the critical process in solving these kinds of puzzles is being able to decompose an item into its more basic components (Knoblich et al., 1999). Whereas chunking refers to binding or grouping perceptual elements for improving efficiency of memory (Miller, 1956), chunk decomposition refers to breaking up a percept into simpler and simpler “chunks” or elements, for reorganization into different compositions (Knoblich et al., 1999, 2001; Luo et al., 2006; Wu et al., 2009, 2013). An element is a perceptual chunk if it has a meaningful perceptual pattern that can be automatically processed, otherwise it is a non-chunk (Knoblich et al., 1999).

The chunk decomposition hypothesis emphasizes the role of the to-be-removed element type as a source of difficulty. The account distinguishes between two kinds of chunks: tight chunks and loose chunks. The decomposition of a tight chunk involves the removal of a perceptual element that carries less meaning (is not a chunk in itself); whereas loose chunk decomposition involves the removal of an element that has a meaningful perceptual pattern (is a chunk on its own). Relative to an element that is a chunk, an element that is a non-chunk is meaningless and is thus harder to identify as an independent element. This triggers an initial mental representation that has a low probability of leading to the solution, causing the difficulty or the impasse (Knoblich et al., 1999, 2001). For example to solve the problem where one must make the equation “XI = III + III” valid by moving one element, the meaningless (non-chunk) element “/” (or “\”) has to be removed from the tight chunk “X” and placed adjacent in order to regroup the “\” and “/” elements into the chunk “V,” forming the valid equation “VI = III + III.” By contrast, when performing the same task on the equation “VII = II + III,” it is easier to remove the meaningful element “I” from the loose chunk “VII” in order to place it with “II” to make the valid equation “VI = III + III.”

However, this account ignores a second potential source of difficulty in chunk decomposition that arises from structural aspects of the stimuli. Namely, the elements of “/” and “\” in the tight chunk “X” are not only of non-chunk type but also crossed, or connected. In contrast, the elements of “V” and “I” in the loose chunk “VI” share both meaningful features and independent space: they are not crossed. Accordingly, the question arises: is the difficulty of chunk decomposition caused by element type or by crossed relation in spatial structure? The chunk decomposition hypothesis has confounded these two sources of difficulty in previous studies (e.g., Knoblich et al., 1999, 2001; Luo et al., 2006; Wu et al., 2009, 2013).

In the current study, we aimed to dissociate the two sources of difficulty (element type: chunk vs. non-chunk; spatial relation: crossed vs. non-crossed) in chunk decomposition. To address this issue, we employed a Chinese character transformation task in which some components have to be removed from one character to another in order to form two new characters (Luo et al., 2006; Wu et al., 2009). This task aligns with the matchstick task with which the chunk decomposition hypothesis has previously been supported (Knoblich et al., 1999, 2001; Luo et al., 2006; Öllinger et al., 2006; Öllinger and Knoblich, 2009; Wu et al., 2009). In addition, like the Roman numerals used in the matchstick task, Chinese characters are perceptual chunks (Tan et al., 2001, 2005; Fu et al., 2002; Siok et al., 2004; Luo et al., 2006; Wu et al., 2009, 2013). What is more, this task can be utilized to dissociate the relative contributions of element type and crossed relation to the difficulty of chunk decomposition, as described below.

**FIGURE 1:**
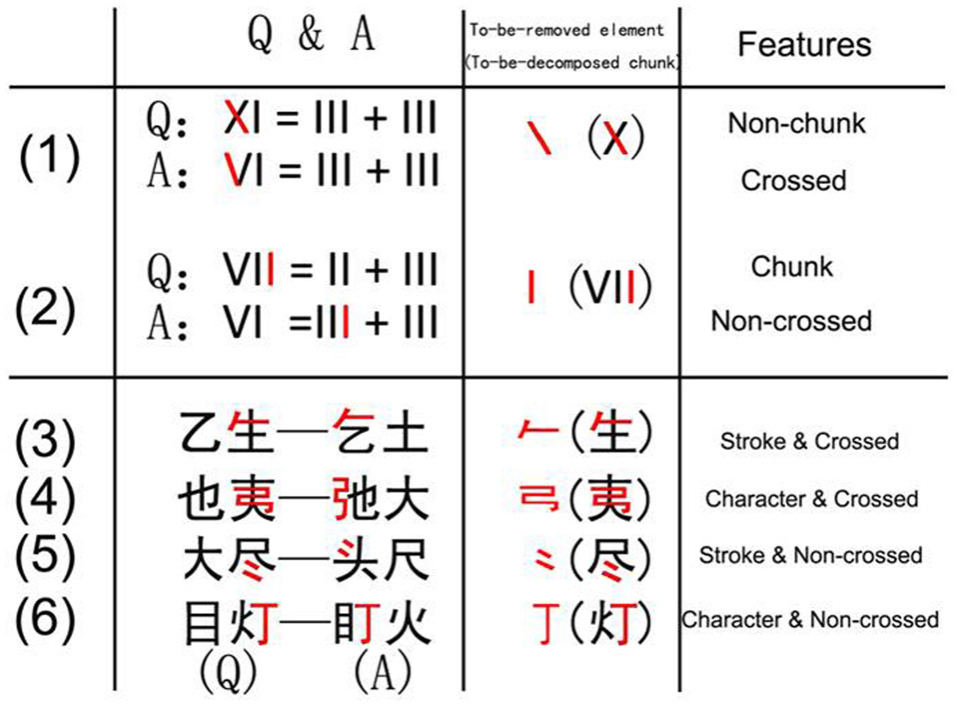
**The Chinese character transformation task maps onto the matchstick arithmetic task used by Knoblich et al. (1999).** “*Q*” denotes question, “*A*” denotes answer. All the to-be-removed elements are marked in red to give a clear description to the reader (Note that in the task all the stimuli are black). The matchstick arithmetic task includes example (1) and (2) and the Chinese character transformation task includes four other examples. In example (1), the to-be-removed element is a non-chunk and has a crossed relation with the remaining element. In example (2), the to-be-removed element is a chunk, and has a non-crossed relation. In example (3), the to-be-removed element is a non-chunk and has a crossed relation. In example (4), the to-be-removed element is a chunk character type and has a crossed relation. In example (5), the to-be-removed element is a non-chunk and has a non-crossed relation. In example (6), the to-be-removed element is a chunk and has a non-crossed relation.

In the Chinese character transformation task (**Figure 1**), an element can be separated from the source character (to-be-decomposed character) as either a character or a stroke. Characters are meaningful perceptual patterns that always carry semantic and phonetic information (like the letters “O” and “R” in the string “OR”). In contrast, strokes are basic components of a Chinese character, carrying considerably less meaning on their own (like the strokes “/” and “\” in the symbol “X”; Luo et al., 2006; Wu et al., 2013). Thus, characters are meaningful chunks whereas strokes are not. According to the chunk decomposition hypothesis (Knoblich et al., 1999, 2001; Luo et al., 2006; Öllinger et al., 2006; Öllinger and Knoblich, 2009; Wu et al., 2009), the transformation task should be relatively easy when the to-be-removed elements are characters. For example, it would be easy to remove the element 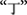 from the chunk 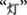 because 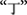 is a character with a meaningful perceptual pattern, whereas the transformation task should be more difficult when one must remove the element 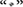 from the character 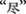 because the element 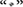 is a basic stroke, a visual unit that carries almost no meaning. In contrast, we hold that a second source of difficulty comes from the structural relation between the elements. Specifically, the removed element can have a crossed or a non-crossed relation with the remaining elements in the source character irrespective of the element type (chunk/character vs. non-chunk/stroke). For example, the chunk 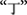 and the chunk 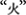 are not crossed with each other in the source character 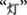 and similarly, the non-chunk 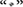 and the chunk 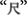 are not crossed in the source character 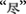 By contrast, the chunks 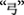 and 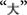 are crossed with each other in the source character 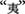 and so are the non-chunk 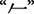 and the chunk 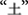 in the source character 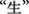 (note that the remaining elements always compose a chunk, but the removed elements can either be a chunk or a non-chunk).

We make two primary predictions with this task. First, performance on the transformation task [as measured by successful completions and response times (RTs)] will be worse when solutions require non-chunk/stroke removals rather than chunk/character removals. Second, solutions requiring crossed-relation removals will be more difficult to arrive at than solutions requiring non-crossed removals. Such results will support our hypothesis that crossed relation plays a significant, and hitherto ignored, role in generating difficulty during chunk decomposition.

## Materials and Methods

### Participants

Thirty paid participants (15 males; age between 21 and 27, mean 24.07 + 1.51 years) participated in the task. They were all native Chinese speakers. All participants had normal or corrected-to-normal vision. This study was approved by the Academic Ethics Committee of Liaoning Normal University. All the participants signed the informed consent and got proper reward for their participation.

### Stimuli

The combination of the two factors (element type vs. crossed relation) gave rise to four conditions in the Chinese character transformation task (see **Table 1**).

**Table 1:** Examples of the Chinese character transformation task in four conditions.

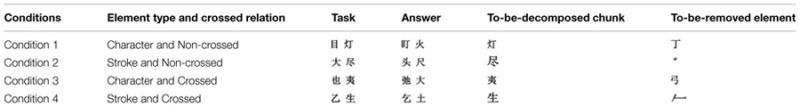

We created 24 pairs of Chinese characters with six examples from each of the four conditions. All characters (including both the original characters and the newly produced characters) were highly familiar to the native Chinese speakers. Eight additional pairs were created for a practice session. The average stroke numbers of the to-be-decomposed characters in the four conditions were 6.50, 6.33, 5.33, and 5.17, for conditions one to four, respectively. The average stroke numbers of the to-be-removed parts of the characters in conditions one to four were 2.67, 2.50, 2.17, and 1.50, respectively.

### Procedure

Participants were seated in a quiet room and tested individually. Each participant received all pairs of characters which were presented in pseudo-random way, with no more than two pairs of the same condition presented consecutively. Two characters were presented simultaneously on the screen for 20 s, with one on the left side and the other on the right side. Participants were instructed to select a part of the character on the right side, and move it to the left character in order to form two new characters according to the following rules (Luo et al., 2006): First, any part of the right-side character could be moved: the to-be-removed part could be characters, strokes, or parts of strokes; Second, no part of the characters could be discarded completely – only moved; Third, the two new characters would have to be valid. Once they found a solution (two new characters), participants were asked to press “1” on the keyboard with their right index finger as quickly as possible and input the answer into the box. If the participants did not find the solution within 20 s, the trial would end automatically. The solution rates and RTs were recorded.

## Results

### Solution Rates and Response Times Analysis

**FIGURE 2:**
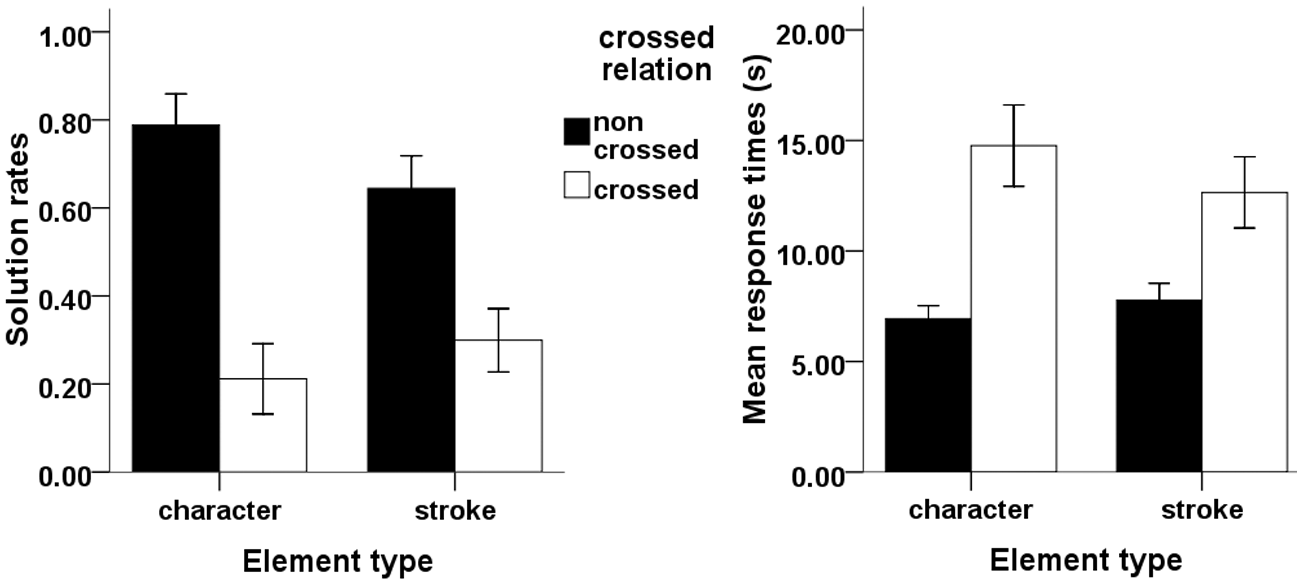
**Illustration of element type effect and crossed relation effect on solution rates **(left)** and mean response times (RTs) **(right)**.** Error bars denote 95% Confidence Interval.

The effects of element type and crossed relation on solution rates are depicted in **Figure 2** (left panel). A 2 × 2 ANOVA on solution rates values with element type (character vs. stroke) and crossed relation (non-crossed vs. crossed) as repeated factors showed a significant effect of crossed relation such that it was more difficult to remove an element that was crossed with the remaining element than a non-crossed element, *F*(1,29) = 183.48, *p* < 0.001, $\eta_{p}^{2}$ = 0.86. The main effect of element type was not significant [*F*(1,29) = 0.96, *p* = 0.34, $\eta_{p}^{2}$ = 0.03]. The interaction of element type and crossed relation was also significant, *F*(1,29) = 17.23, *p* < 0.001, $\eta_{p}^{2}$ = 0.37. For non-crossed relations, strokes were more difficult to remove than characters, *F*(1,29) = 9.64, *p* = 0.004, $\eta_{p}^{2}$ = 0.25, whereas for crossed relations, characters were more difficult to remove than strokes, *F*(1,29) = 7.24, *p* = 0.012, $\eta_{p}^{2}$ = 0.21).

There were 120 data points in total (30 participants multiplying by four conditions). For 16 out of the 120 cases, no RTs were recorded because they did not solve any problems in a given condition. In these cases, the missing RT values were recorded as 20 s (the time limit for providing an answer) in each condition (11 in condition 2 and 5 in condition 4, respectively). The average RTs are depicted in **Figure 2** (right panel). A 2 × 2 ANOVA on RT with element type (character vs. stroke) and crossed relation (non-crossed vs. crossed) as repeated factors showed a significant main effect of crossed relation [*F*(1,29) = 74.73, *p* < 0.001, $\eta_{p}^{2}$ = 0.72] but not of element type (*F* = 1.67, *p* = 0.21, $\eta_{p}^{2}$ = 0.05). This indicates that solutions took longer in the crossed condition than in the non-crossed condition. The interaction of element type and crossed relation was also significant [*F*(1,29) = 6.98, *p* = 0.013, $\eta_{p}^{2}$ = 0.19]. The simple effect of element type in the non-crossed relation condition was significant [*F*(1,29) = 5.74, *p* = 0.023, $\eta_{p}^{2}$ = 0.17], indicating that stroke solutions took longer to find than character solutions in the non-crossed condition. The simple effect of element type in the crossed relation condition was significant as well [*F*(1,29) = 4.53, *p* = 0.042, $\eta_{p}^{2}$ = 0.14], indicating that character solutions took longer to find than stroke solutions in the crossed condition.

### Bivariate Correlation Analysis Based on the Tasks

There were 24 Chinese character transformation task problems in total. For each problem, we assessed two additional, very basic characteristics that might influence problem difficulty: the number of strokes that composed the to-be-removed element and the number of strokes that composed the source character. In addition, we also assessed whether perceptual crossings (the number of crossed dots where the to-be-moved element crossed with the remaining element. For example there were three crossed points between the to-be-removed element 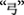 and the remaining element 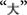 in the case of 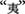) influence problem difficulty in the 12 transformation tasks with elements in crossed relationship. We used a bivariate correlation analysis (Pearson’s *r*) to determine how these factors impacted solution rates and RT independently. None significant correlations were found between any of the three factors and solution rates or RT. For strokes number composing the to-be-removed elements: *r*(24) = 0.277, *p* > 0.05 and *r*(24) = -0.387, *p* > 0.05, respectively. For strokes number composing the source character: *r*(24) = 0.048, *p* > 0.05 and *r*(24) = -0.002, *p* > 0.05, respectively. For perceptual crossings: *r*(12) = 0.032, *p* > 0.05 and *r*(12) = -0.344, *p* > 0.05, respectively.

## Discussion

The goal of this study was to expose a potential confound in experiments involving chunk decomposition. Whereas previous studies have ignored this potential source of difficulty (Knoblich et al., 1999, 2001; Luo et al., 2006; Wu et al., 2009, 2013), we found that crossed vs. non-crossed relation is a relatively more influential factor impacting the difficulty of chunk decomposition. It is important to note that our findings do not invalidate the previous work cited above, as we have replicated the finding that element type impacts chunk decomposition difficulty. However, we do show that this finding is limited to our non-crossed condition. In our crossed condition the effect was in the opposite direction.

The results indicate that the effect of crossed relation on chunk decomposition difficulty is robust: regardless of element type, crossed relations caused lower solution rates, and higher RTs than non-crossed relations. Why does a crossed relationship make it so difficult to decompose? One possible explanation is the global precedence effect, according to which the perceptual response to a global shape is faster than to a local shape and the influence of the global shape on the local shape is stronger than the reverse influence (Navon, 1977). In a chunk decomposition task (e.g., the matchstick problem, or the Chinese character transformation task), a holistic processing of the to-be-decomposed perceptual chunk can be formed automatically during the first exposure to the problem. This can cause a very stable perceptual set that biases the perceiver against recognizing the component chunks. In the current study, the Chinese character is a perceptual chunk processed holistically (e.g., Chen et al., 2013), which gives rise to a perceptual set automatically, both in the crossed condition and the non-crossed condition. Relative to non-crossed relations, we speculate that crossed relationships strengthen the global effect, making the identification of a component element more difficult.

A second explanation of our effects is that the crossed relation might cause chunk decomposition difficulty in a manner related to pattern masking (Enns and Di Lollo, 2000). In pattern masking, the acuity of a visual target is reduced by the presentation of another spatially superimposed contour (Enns and Di Lollo, 2000). The elements composing our stimuli were not completely masked by one another. However, some partial pattern masking may have occurred in the crossed relation condition, decreasing the salience of the target element.

## Conclusion

In short, the roles of element type and crossed relation in generating difficulty during chunk decomposition were dissociated in this study. Significant effects of both factors were demonstrated, however, the effect of crossed relation was larger and more consistent than the effect of element type. We conclude that crossed relation is a more fundamental source of difficulty in solving perceptual problems that involve chunk decomposition than element type, and recommend that this source of difficulty should be taken into account in future experiments.

## Conflict of Interest Statement

The authors declare that the research was conducted in the absence of any commercial or financial relationships that could be construed as a potential conflict of interest.

## References

[B1] Chen H. Bukach C. M. Wong A. C-N. (2013). Early electrophysiological basis of experience-associated holistic processing of Chinese characters. *PLoS ONE* 8:e61221 10.1371/journal.pone.0061221 PMC3623809 23593436

[B2] Cranford E. A. Moss J. (2012). Is insight always the same? A protocol analysis of insight in compound remote associate problems. *J. Probl. Solving* 4 128–153. 10.7771/1932-6246.1129

[B3] Enns J. T. Di Lollo V. (2000). What’s new in visual masking?*Trends*. *Cogn. Sci.* 4 345–352. 10.1016/S1364-6613(00)01520-5 10962616

[B4] Fu S. Chen Y. Smith S. Iversen S. Matthews P. M. (2002). Effects of word form on brain processing of written Chinese. *Neuroimage* 17 1538–1548. 10.1006/nimg.2002.1155 12414292

[B5] Knoblich G. Ohlsson S. Haider H. Rhenius D. (1999). Constraint relaxation and chunk decomposition in insight problem solving. *J. Exp. Psychol. Hum. Learn.* 25 1534–1555. 10.1037/0278-7393.25.6.1534

[B6] Knoblich G. Ohlsson S. Raney G. (2001). An eye movement study of insight problem solving. *Mem. Cognit.* 29 1000–1009. 10.3758/BF03195762 11820744

[B7] Kounios J. Beeman M. (2014). The cognitive neuroscience of insight. *Annu. Rev. Psychol.* 65 71–93. 10.1146/annurev-psych-010213-115154 24405359

[B8] Luo J. Niki K. Knoblich G. (2006). Perceptual contributions to problem solving: chunk decomposition of Chinese characters. *Brain Res. Bull.* 70 430–443. 10.1016/j.brainresbull.2006.07.005 17027779

[B9] Miller G. A. (1956). The magical number seven plus or minus two: some limits on our capacity for processing information. *Psychol. Rev.* 63 81–96. 10.1037/h0043158 13310704

[B10] Navon D. (1977). Forest before trees: the precedence of global features in visual perception. *Cogn. Psychol.* 9 353–383. 10.1016/0010-0285(77)90012-3

[B11] Ohlsson S. (1984). Restructuring revisited: II. An information processing theory of restructuring and insight. *Scand. J. Psychol.* 25 117–129. 10.1111/j.1467-9450.1984.tb01005.x

[B12] Öllinger M. Jones G. Knoblich G. (2006). Heuristics and representational change in two-move matchstick arithmetic tasks. *Adv. Cogn. Psychol.* 2 239–253. 10.2478/v10053-008-0059-3

[B13] Öllinger M. Knoblich G. (2009). “Psychological research on insight problem solving,” in *Recasting Reality* eds Atmanspacher H. Primas H. (Berlin-Heidelberg: Springer) 275–300. 10.1007/978-3-540-85198-1_14

[B14] Siok W. T. Perfetti C. A. Jin Z. Tan L. H. (2004). Biological abnormality of impaired reading is constrained by culture. *Nature* 431 71–76. 10.1038/nature02865 15343334

[B15] Tan L. H. Laird A. R. Li K. Fox P. T. (2005). Neuroanatomical correlates of phonological processing of Chinese characters and alphabetic words: a meta-analysis. *Hum. Brain Mapp.* 25 83–91. 10.1002/hbm.20134 15846817 PMC6871734

[B16] Tan L. H. Liu H. L. Perfetti C. A. Spinks J. A. Fox P. T. Gao J. H. (2001). The neural system underlying Chinese logograph reading. *Neuroimage* 13 836–846. 10.1002/hbm.20134 11304080

[B17] Wu L. L. Knoblich G. Luo J. (2013). The role chunk tightness and chunk familiarity in problem solving: evidence from ERPs and fMRI. *Hum. Brain Mapp.* 34 1173–1186. 10.1002/hbm.21501 22328466 PMC6870504

[B18] Wu L. L. Knoblich G. Wei G. X. Luo J. (2009). How perceptual processes help to generate new meaning: an EEG study of chunk decomposition in Chinese characters. *Brain Res*. 1296 104–112. 10.1016/j.brainres.2009.08.023 19695234

